# Development of a Portable Non-Invasive Swallowing and Respiration Assessment Device †

**DOI:** 10.3390/s150612428

**Published:** 2015-05-27

**Authors:** Wann-Yun Shieh, Chin-Man Wang, Chia-Shuo Chang

**Affiliations:** 1 Department of Computer Science and Information Engineering, Chang Gung University, No. 259, Wen-Hwa 1st Road, Kwei-Shan, Tao-Yuan 333, Taiwan; E-Mail: johnny7802@hotmail.com; 2 Department of Physical Medicine and Rehabilitation, Chang Gung Memorial Hospital, Taoyuan 333, Taiwan; E-Mail: cmw1314@cgmh.org.tw; 3 Department of Medicine, Chang Gung University, No. 5, Fu-Hsing Street, Kwei Shan, Tao-Yuan 333, Taiwan

**Keywords:** swallowing, respiration, dysphagia, thyroid cartilage motion, non-invasive detection, FSR

## Abstract

Dysphagia is a condition that happens when a person cannot smoothly swallow food from the mouth to the stomach. It causes malnourishment in patients, or can even cause death due to aspiration pneumonia. Recently, more and more researchers have focused their attention on the importance of swallowing and respiration coordination, and the use of non-invasive assessment systems has become a hot research trend. In this study, we aimed to integrate the timing and pattern monitoring of respiration and swallowing by using a portable and non-invasive approach which can be applied at the bedside in hospitals or institutions, or in a home environment. In this approach, we use a force sensing resistor (FSR) to detect the motions of the thyroid cartilage in the pharyngeal phase. We also use the surface electromyography (sEMG) to detect the contraction of the submental muscle in the oral phase, and a nasal cannula to detect nasal airflow for respiration monitoring during the swallowing process. All signals are received and processed for swallowing event recognition. A total of 19 volunteers participated in the testing and over 57 measurements were made. The results show that the proposed approach can effectively distinguish the swallowing function in people of different ages and genders.

## 1. Introduction

The difficulty in swallowing food through the esophagus to the stomach, is a condition known as dysphagia. Many acute or chronic illnesses may cause dysphagia, majorly of two types: (I) structural dysphagia, when a structural tissue abnormality like in a cleft lip and palate born child, or oral cancer affects the swallowing function; (II) nervous system disorder, when some nervous system abnormality such as cerebral palsy, myasthenia gravis, stroke or another disease causes a swallowing dysfunction (for stroke abnormalities, the proportion of the patients with the oropharyngeal dysphagia may reach 32% to 45% [1])

The study of Greco *et al.* [1] showed that over 12% of patients in an acute medical institute had dysphagia, while in a long-term healthcare organization, the proportion may be more than 50%. If the symptoms of oropharyngeal dysphagia are not properly treated, it can lead to many complications. The most important one is that the eating behavior of patients will be affected and changed, which leads to dehydration, malnutrition, choking injuries, aspiration pneumonia and even death [2,3,4]. Typically, the swallowing process can be divided into four stages:

(1) Oral preparation stage: the mouth starts to chew the food and mix it with saliva.
(2) Oral stage: the bolus is pushed backwards by the tongue into the pharynx.
(3) Pharyngeal stage: many swallowing reflex motions occur, including a series of complex throat neuromuscular reactions, which can push the bolus into the esophagus.
(4) Esophagus stage: the bolus is pushed into the stomach through the esophagus.

Dysphagia can happen at any one of above stages, but the general assessment and therapy for neurogenic dysphagia particularly emphasizes the oral and the pharyngeal stage. This is because the entrance of the esophagus is in the close proximity to the larynx and both air and the swallowed bolus will share a common pathway through the pharynx. Many researchers have mentioned that breathing and swallowing are physiologically linked to ensure smooth gas exchange during oronasal breathing, and to prevent aspiration during swallowing [2,5,6,7,8]. When the food goes through the pharynx to the esophagus, swallowing and respiration cannot happen at the same time. This physiological mechanism is to ensure that the food can be swallowed through the esophagus smoothly and safely without getting into the trachea and lungs. It can also avoid suffocation, aspiration pneumonia and severe respiratory failure. In fact, most of the oropharyngeal dysphagia cases actually result from uncoordinated respiration and swallowing in the oropharyngeal stage. Therefore, bedside clinical evaluation is very important. Currently most oropharyngeal dysphagia evaluations are performed by well-trained physicians or speech therapists. A physician will ask the subjects to swallow food or liquid, and the physician record the strength and the speed of vomiting, coughing, swallowing reflex motions, eating posture, the difficulty of eating different kinds of food and nutritional status, *etc.*

Most instrumental methods for measuring oropharyngeal dysphagia symptoms are invasive approaches. The most widely used method is the video fluoroscopic swallowing study (VFSS), which is the gold standard of the oropharyngeal dysphagia examination in many medical organizations [9,10]. It uses an X-ray photography instrument. Before the test, the physician will ask the subject to swallow food containing a contrast material. By observing the distribution of the remains of the contrast material in the mouth and throat, the physician can adjust the degree of the oropharyngeal dysphagia. It is an accurate method. However, it will bring a risk of radiation exposure.

The second instrumental method uses a fiber optic endoscope to evaluate oropharyngeal dysphagia [11]. A subject will be asked to eat a colorful food or liquid, then the physician will use an endoscope with optical fiber to observe the motion of the nasopharynx in the mouth and throat. This approach can help the physician observe the reflected swallowing flow in the rest state of the oropharynx and hypopharynx before and after the subject eats the food. However, this method needs an endoscope placed in the mouth and throat during the test, making the subject feel uncomfortable and swallowing the food difficult.

Both of the above approaches require the patients to undergo the testing in a hospital or lab. Thus, the measurements will be limited in a specific time and place. For patients with poor mobility, it is very inconvenient to do the testing with the risk of radioactive exposure or nosocomial infection. Therefore there is a strong need to develop a wearable and portable device, as well as the corresponding assessment software, so physicians can perform the measurements directly near the bedside.

**Figure 1:**
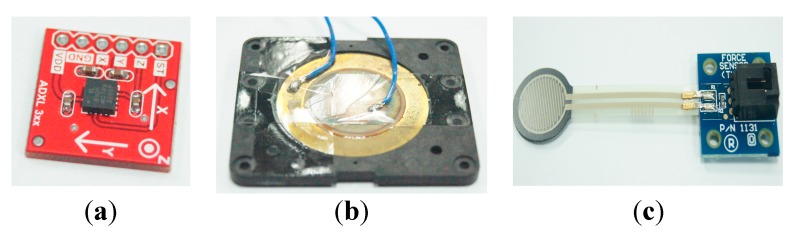
Micro-Electro-Mechanical sensors: (**a**) accelerometer; (**b**) piezoelectric sensors; (**c**) force sensing resistor.

Some researchers have applied the Micro-Electro-Mechanical Systems (MEMs) sensors to measure oropharyngeal dysphagia [5,12]. The most widely used sensors for this purpose are accelerometers and pressure sensors, especially those based on the piezoelectric effect. We take the sensor in Figure 1a for example. Figure 1a shows a three-axis capacitive accelerometer which can perform static or dynamic acceleration measurements. The study of Lee *et al.* [12] pasted an accelerometer on a subject’s throat to measure the upward and downward motions of the thyroid cartilage when the subject swallowed water (or food). The studies reported in [1,5,12] proposed similar approaches. Using an accelerometer, however, has an obvious drawback. That is, the measurement results will suffer signal interference from the subject’s head or trunk shaking. Although there are other accelerometers which only measure vibrations, they cannot measure the force exerted by the thyroid cartilage.

The piezoelectric sensor (shown in Figure 1b) is the kind of pressure sensors used in [13]. It is made by solid materials, which can accumulate electric charges in response to applied mechanical stress. The authors pasted a piezoelectric sensor tightly on a subject’s throat. When a subject swallows water or food, the thyroid cartilage will press the sensor, making the sensor respond with a voltage. The voltage can be translated into a pressure, and a physician can determine the force exerted by the thyroid cartilage on the sensor from that pressure. Most piezoelectric sensors, however, are inflexible materials, which means that a subject may feel uncomfortable wearing them on the skin surface of the thyroid cartilage. In addition, pasting a solid sensor on the throat will affect a subject’s swallowing function, which causes measurement bias.

There is a type of sensor which was not used in previous research, the force-sensing resistor (FSR), shown in Figure 1c. It is a kind of piezoresistive sensor. Its use principle requires contact with both sides of the sensor such that its resistance will be changed. Applying a larger pressure on both sides of the FSR will cause better conductivity, therefore we can connect a small power source to the sensor and detect a force applied on the sensor by measuring changes in the output voltage. Because the FSR can be manufactured into a very small thin-film structure, it is often used to measure the foot pressure [14,15]. There are advantages if we use a FSR sensor to detect the thyroid cartilage motions in the pharyngeal stage. First, it is a soft and flexible material. Thus we can paste the FSR sensor on the throat without affecting the swallowing function. Second, we can measure the output voltages of the sensor to monitor the force the throat exerts on the surface of the sensor during the swallowing. This can help us to develop an easy-to-use, lightweight, and wearable belt device for healthcare applications or bedside assessment.

**Figure 2:**
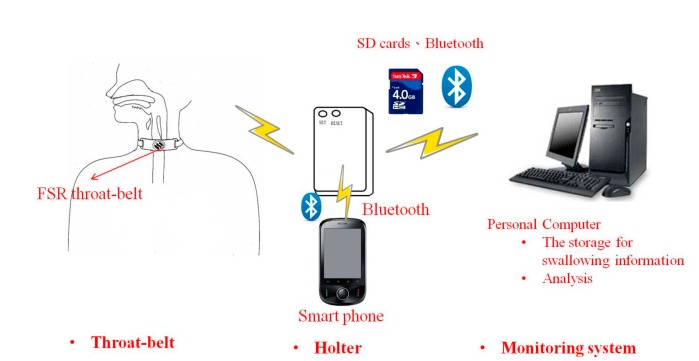
The diagram of the wearable swallowing monitoring system.

This paper aims to use the FSR to design a wearable and portable monitoring system for oropharyngeal swallowing. Such a system can be applied to bedside assessments or homecare applications. The diagram of this system is illustrated in Figure 2. The system consists of a FSR throat belt, a holter and a remote monitoring system. The FSR throat belt is made by soft and flexible materials with a FSR embedded in the middle of the belt. The signals measured by the FSR will be collected in the holter. The holter performs basic signal processing with a 1 kHz sample rate, 20 Hz low-pass filtering, and 8-bit analog-to-digital (A/D), *etc.* The data afterwards can be displayed immediately on a smart phone or be sent to a remote monitoring system for further physiological analysis. Later we will show the detailed design of the FSR belt and its test results. An implementation example of the holter system will be shown before the end of this paper.

The concept of this work was first reported in [16] with less than 10% overlap with the current pape, where we clarify the design in more details and include more test results. The remainder of this paper is organized as follows: in Section 2 we survey the previous research on approaches for monitoring dysphagia. In Section 3 we present the design of the proposed FSR belt and the signal analysis method. In Section 4 we first present the testing method, and then the test results with discussion. Finally, Section 5 gives the conclusions.

## 2. Related Works

Many researchers have mentioned that the evaluation of the swallowing function before treatment is very important. According to the studies in [2,3,4], a patient’s quality of life can be greatly affected by dysphagia. To observe swallowing behavior, Logemann *et al.* [10] used VFSS equipment to monitor the swallowing motion during the oral and pharyngeal stage. Ertekin and Pehlivan *et al.* combined the electrophysiological and mechanical approaches to measure the laryngeal motions with submental sEMG during swallowing [3]. They proved that the study of the physiology of deglutition is very useful in the clinical evaluation of dysphagia patients.

Some researchers [17,18,19] have mentioned that the tongue pressure will also affect the swallowing function. They designed an apparatus based on a three-bulb silicon array to measure the tongue pressure. The apparatus is a handheld device, and can be put into fixed positions above the tongue for measurement. When the subject’s tongue touches the silicon array, each bulb will reflect the pressure during the swallowing process. This approach, however, cannot reflect the swallowing status in the pharyngeal stage.

Martin-Harris and Brodsky [6,7] developed a normative model to integrate breathing and swallowing patterns, using concomitant videofluoroscopic images and nasal respiratory airflow recordings. Catiuscia and Luiz have mentioned that the methods currently used for diagnosis, however, are only qualitative, and need expensive equipment [1]. Also, most current clinical bedside evaluations are based on physician experience. Therefore, in Catiuscia and Luiz’s study [1], they used the three-axis accelerometer to measure the neck vibrations associated with deglutition, and used a PDA as a portable device for bedside measurements. Guilherme and Evert *et al.* [5] used the nasal airflow measurement subsystem with the accelerometers to measure the correlations between breathing and swallowing.

There are other researches which evaluated how different factors of bolus affect the swallowing physiology, including the temperature or the volume [13]. From the research in [13], we find that if the volume of water was larger than 20 mL, some subjects failed to swallow the bolus after the first try and they had to divide the bolus into two or more pieces as piecemeal deglutition in a hotter temperature range.

In our approach, we use not only the FSR throat-belt to measure swallowing motions, but also a nasal airflow sensor to monitor the association with respiration. By integrating the signals from the FSR throat-belt and the nasal airflow sensor, we can compare the correlations between swallowing and breathing. Moreover, we have developed a portable holter to continuously collect the data. The data can be transmitted to computers via wireless communication, making our approach is very suitable for bedside and homecare measurement applications.

## 3. Methods

In this section we show the design of the proposed FSR throat belt. Then we present the algorithm by which the swallowing and breathing events can be identified automatically from different signals (*i.e.*, FSR, sEMG, and nasal airflow).

### 3.1. FSR Throat-Belt Design

**Figure 3:**
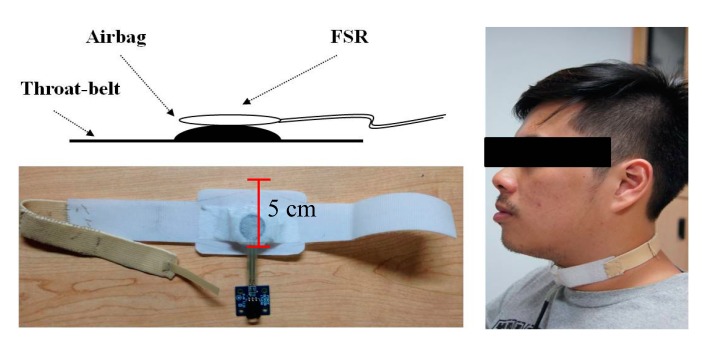
The FSR throat-belt.

**Figure 4:**
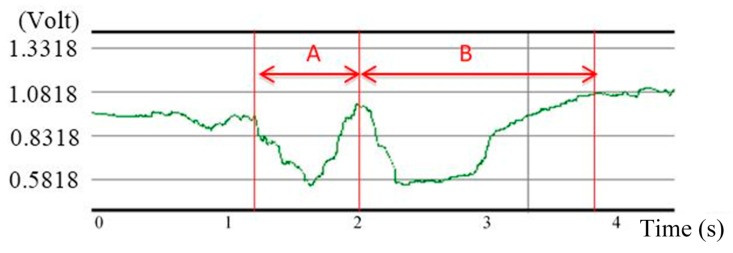
A typical swallowing signal measured by FSR on a healthy subject.

The major component of this work is the FSR throat-belt design. Recall that the FSR should be used by contacting both sides of the sensor to change the internal conductivity. We found that if we only use medical tape to paste the FSR on a subject’s throat, the signals could not be obviously detected because medical tape cannot provide a sufficient reaction force on the FSR as the larynx or the thyroid cartilage does during swallowing. To resolve this problem, we design a throat-belt where the FSR sensor is fixed on the center of the belt and the subject can wear it around the neck, as shown in Figure 3. The belt has good elasticity and we use Velcro straps to close it. The maximal width of the belt is 5 cm, therefore it does not obstruct the natural swallowing motions. Particularly, we insert a small airbag between the belt and FSR such that FSR can be fixed on the center of the thyroid cartilage without any movement during the testing. If FSR is not fixed on that point, the measurement accuracy will be affected. When the belt is tied around the neck, the airbag will provide a stable initial pressure on the FSR which will be considered as the baseline in each measurement. During swallowing, the larynx and the thyroid cartilage will retract, resulting in released and changed pressure on the FSR, as shown in Figure 4.

We let a healthy subject (21 years old) swallow 10 mL of room temperature water as an example. Figure 4 shows the swallowing signal measured by FSR. At first, the FSR detected the initial pressure from the airbag. Then two significant responses followed during time periods “A” and “B”. The first valley in time period “A” represents that the larynx starting to move upward and forward such that the bolus can be pushed on the top of the esophagus. After that, the second valley in time period “B” appears, representing when the thyroid cartilage retracts and the bolus is pushed into the esophagus. Finally the thyroid cartilage returns back to the original position. Because the belt is made of all soft materials, such results can reflect real motions of the thyroid cartilage without affecting the subject’s swallowing function.

### 3.2. Signal Analysis for the Physiology of Swallowing

We are interested in finding the correlations between swallowing and respiration. Therefore we attach the FSR belt around the neck, paste a pair of sEMG electrode pads on the submental muscle, and put the nasal airflow cannula in front of the nose. To synchronize those three different signal sources, in the following measurements we connect all of them into a BIOPAC MP100 system for data acquisition and analysis. Also, here we mostly focus on detecting the timing of swallowing events. We do not calibrate the voltage domain (Volts) of signals to the force domain (N); *i.e.*, we only display the voltages of signals. Later we will discuss the implementation of the holter (*cf.* Figure 2) such that the FSR belt and other signals can be collected for practical applications.

**Figure 5:**
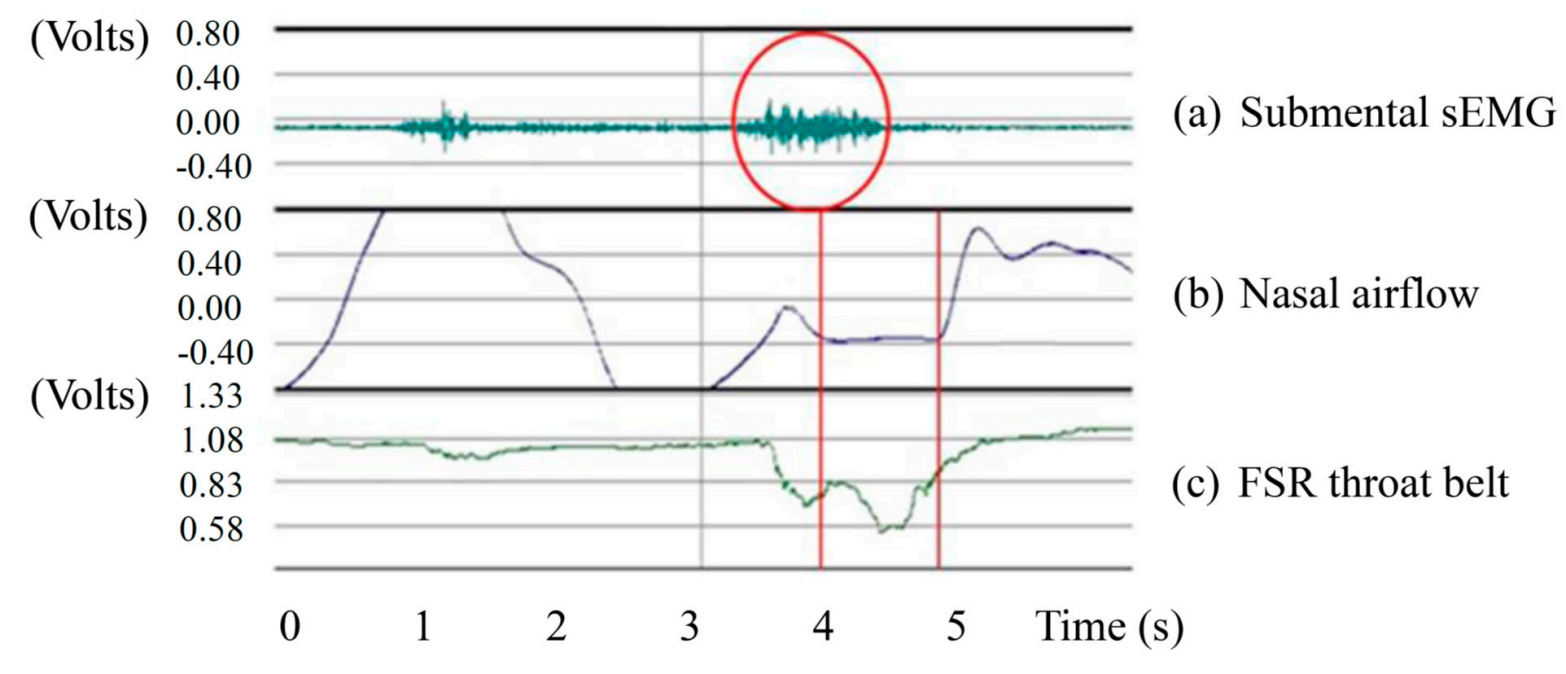
Signals from three sensors: (**a**) Submental sEMG; (**b**) Nasal airflow; and (**c**) FSR throat belt (the Y-axis preserves the original scaling in the BIOPAC MP100 for each signal).

We use the sEMG sensor to detect the signals from the surface of the submental muscle. Figure 5a,c show the submental sEMG and the FSR signals from a healthy subject swallowing 10 mL of room temperature water. The sEMG signals have been amplified by 1000 times on a scale between −1 Volts and 1 Volts. In the figure we find that the onset time of sEMG covered most of the swallowing process. This is reasonable because when we swallow the bolus, the submental muscle will continue to squeeze the bolus into the top of esophagus. Thus we can use the responses of the submental sEMG to identify the beginning and the ending of the pharyngeal stage in each swallowing test.

We also used the nasal cannula to measure the airflow changes caused by respiration. The amount of nasal airflow through the nasal cannula will be translated into digital signals by a pressure transducer. Figure 5b,c show the nasal cannula signals and the FSR signals in the same 10 mL room temperature water swallowing test. The respiration of healthy participants, as shown in the figure, will display an apnea episode during the swallowing. It is because our the human physiological mechanism will protect us reflectively from choking injuries. According to the signals from the nasal airflow and the FSR throat-belt, a physician can immediately evaluate the coordination between the swallowing and the respiration.

**Figure 6:**
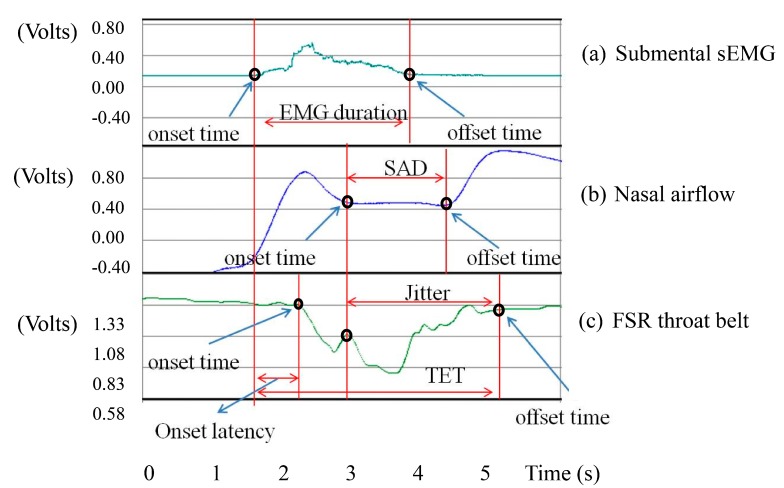
Representative non-invasive swallowing measurements: (**a**) Submental sEMG; (**b**) Nasal airflow; and (**c**) FSR throat belt.

By analyzing the signals from these three sensors (*i.e.*, Figure 5), we can find that the onset time and the offset time of each signal have different physiological meanings, which can be used to partition a normal swallowing process into several periods:

(1) EMG duration: This is the duration of the submental muscle motion, which can be obtained by measuring the time interval between the onset time and the offset time of the submental sEMG waveform, as shown in Figure 6a.
(2) Onset latency: This is the duration from the onset time of the submental muscle “starting to contract”, to the onset time of the thyroid cartilage “starting to move upwards”. It can be obtained by measuring the time interval between those two onset time points of the submental muscle sEMG and the throat-belt waveforms, respectively, as shown in Figure 6a,c.
(3) Total excursion time (TET): This is the time from when the thyroid cartilage starts to move upwards to the thyroid cartilage returning to the original status. It can be obtained by measuring the time interval between the onset time and the offset time of the FSR throat belt waveform, as shown in Figure 6c.
(4) Swallowing apnea duration (SAD): This is the time of apnea, which can be obtained by measuring the time interval between the onset time and the offset time of the nasal airflow waveform, as shown in Figure 6b.
(5) Jitter: The time while the throat is pushing the bolus into the esophagus. It can be obtained by measuring the time interval between the turn-around point and the offset time from the throat belt waveform (*i.e.*, the period “B” in Figure 4), as shown in Figure 6c.

**Figure 7:**
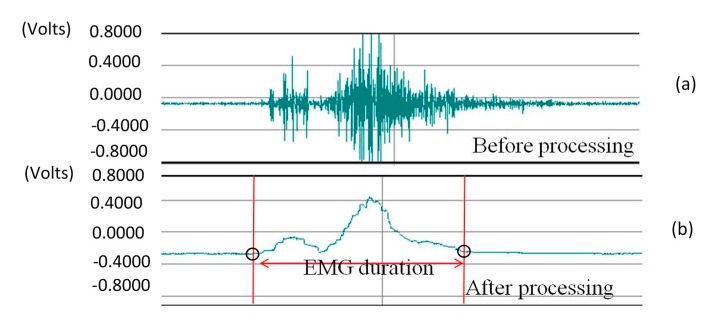
The processing of the submental muscle sEMG: (**a**) Before; (**b**) After.

**Figure 8:**
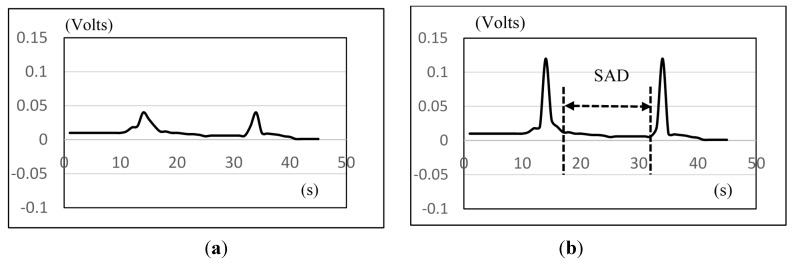
The amplification and the detection of the nasal airflow signals: (**a**) Before; (**b**) After.

The time intervals mentioned above all involve event identification among different signals. In order to identify the timing of those events automatically for analysis, we apply the following pre-processing steps to each signal:

(1) For submental muscle sEMG: We use the BIOPAC MP100 system [20] to record the sEMG signals (e.g., Figure 7a). The sEMG signals were filtered through a Butterworth digital filter (second-order band-pass filter) with a passband of 20–400 Hz, and smoothed using a 15-point moving average [21,22], as shown in Figure 7b. To identify the sEMG onset time, we fetch the data from the first three seconds and calculate the mean (M) with the standard deviation (SD). For the data (say: sEMG[*i*]) after the third second, we set a threshold (*M* + α · *SD*), where α is a factor ranged from 1 to 3 (the value of α should be determind by real signal patterns; in this work, we set the value of α as one.) If sEMG[*i*] is the first point of which the value is over the threshold, we mark it as the onset time point. Typically a swallow will finish in a few seconds. Therefore we fetch totally four-second data after the oneset time point for analysis. If sEMG[*j*] is the last point of which the value is over the threshold in this period, we mark it as the offset time point. The time between sEMG[*i*] and sEMG[*j*] is the “EMG duration”.

(2) Nasal airflow sensor: We also use the BIOPAC MP100 system [20] to record nasal airflow signals (e.g., Figure 8a). In order to enhance the detection accuracy, we differentiate between the inspiration and the expiration phases first. That is, if the data (say nasal[*k*]) is larger than a threshold (*M* + β · *SD*), we amplify the value of nasal[*k*] by *N* times. Here, M is the mean of the first three-second data (baseline), SD is the standard deviation, and in the following testing we let β = 1 and *N* = 3. Figure 8b shows the results after the amplification. Then we can apply the similar method to detect the onset time and the offset time. The interval between both time points is SAD.

(3) Throat-belt: To detect the onset time and the offset time of the FSR signals, we apply the same approach used in the sEMG detection. We calculate the mean of data in the first three seconds as the baseline, and set a threshold by subtracting a ratio of the standard deviation from the mean (*i.e.*, (*M* − *r* · *SD*, 0.5 ≤ *r* ≤ 1), The value of *r* should be determined by real signal patterns (in this work, we set the value of *r* as one). The onset time and the offset time can then be identified as the first and the last points less than the threshold, respectively. Particularly, we need to detect the middle turn-around point between the onset and the offset points for calculating the jitter (*cf.* Figure 6). The turn-around time detection can be done by scanning the slope variation along the curve between the onset and the offset time points.

## 4. Results and Discussion

We used the same equipment described in [21,23] to verify the proposed approach, including the nasal cannula to measure the nasal airflow, the sEMG electrode pad to measure the submental muscle motions, and finally using the BIOPAC MP100 system to record all signals. Instead of using the piezoelectric sensor as in [21,23], in this study we used the proposed FSR throat belt to detect the motions of the thyroid cartilage.

We recruited nineteen healthy subjects to participate in the testing, and divided them into three age groups: 21–30 years old, 31–50 years old and 51–60 years old, as shown in Table 1. The exclusion criteria were any known history of dysphagia, cardiopulmonary disease, neurological disease, hiatal hernia, chronic indigestion disorder, gastroesophageal reflux disease, cancer or disease of the head and neck, current use of medications with known effects on swallowing or breathing, tobacco use in the past 10 years, or age of more than 70 years. We excluded participants aged more than 70 years, because this age group is more likely to have comorbidities that influence swallowing and respiration. The Ethics Committee of Chang Gung Medical Foundation approved this study. All participants signed informed consent forms before participation.

**Table 1:** Characteristics of participants in the three age groups.

Group	Age Range	Mean ± SD	Male	Female
Young	21–30	24 ± 3	4	3
Middle	31–50	37 ± 5	4	4
Old	51–60	55 ± 4	2	2

During the experiment, the participant sat on a chair with a natural posture. We cleaned the surface skin of the jaw and throat using medicinal alcohol. After that we pasted the sEMG electrode on the submental muscle. Also, we helped the participants fit the throat-belt such that the FSR can be placed on the surface of the thyroid cartilage, and the nasal airflow cannula is placed in front of the nose.

**Figure 9:**
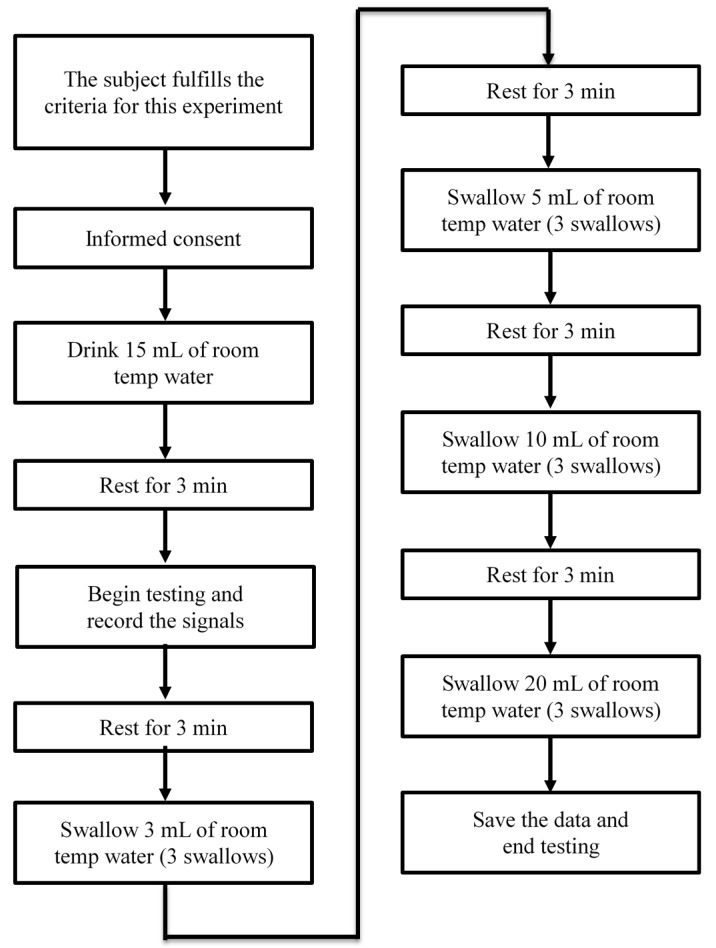
The standard operating procedure in our testing.

The experiment requires consumption of five different volumes (1, 3, 5, 10 and 20 mL) of room temperature water. Each subject performed three trials for each volume. After each trial, we let the subjects rest for three minutes. Most importantly, we remind the subjects to swallow normally and within their ability. The standard operating procedure (SOP) is shown in Figure 9. For safety considerations, we did not ask the participants to do the counterbalancing measurement. We asked the participants to start the test from a small volume of water, and increase the water volume step by step. If we do not know the participant’s swallowing limit in advance and start the test with a large volume of water, he or she may have choking risk which affects the subsequent testing.

### 4.1. Experimental Results

In this section, we will show the results measured by the submental muscle sEMG, the nasal airflow, and the FSR throat belt, as well as the correlation analysis between them. Our goal is to show that the proposed belt can have good sensitivity to the effects of age, the volume of water swallowed, or the gender of the subjects performing the swallowing functions.

#### 4.1.1. The Results of sEMG and Nasal Air Flow

**Figure 10:**
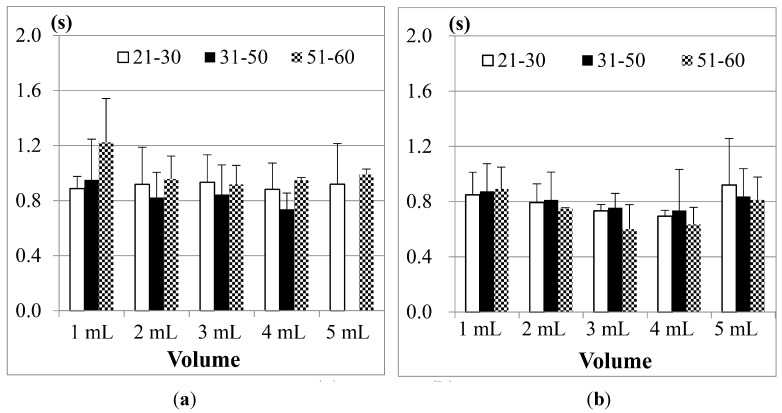
Comparison of the average duration time of the submental sEMG in different age groups for (**a**) male; and (**b**) female participants. The X-axis lists the water volumes and the Y-axis shows the mean duration time.

Figure 10 shows the comparison results of sEMG in different age groups, where (a) is for male participants and (b) is for female participants. The first point we found is, although 1 mL water is the smallest water volume, it does not mean that the participants (no matter whether male or female) needed the least time to swallow it. Also, as the age of the participants increases, the sEMG duration will have a slight increase. This fact was also found in other previous studies [21,23,24]. A more acceptable reason is that 1 mL water is too small a sample so most participants need to use more strength and time to swallow it. The other point is, for 3, 5 or even 10 mL water, there are no obvious differences among different age groups. This result shows that using sEMG alone would not be a good approach to evaluate the swallowing function, because the duration time of sEMG actually cannot represent the whole swallowing process (however, in some previous studies, like [18,24], they evaluated the swallowing function by only observing the strength or the duration of sEMG).

Note that the results of the 31 to 50 years old male group in the 20 mL trials are not included in Figure 10a. This is because the participants in this group peformed piecemeal swallowing (also known as “piecemeal deglutition” [13]), where they divided the water into smaller volumes, and finished the swallowing in several times. In this case, the volume represents a dysphagia limit and the duration time of swallowing fluctuates, which makes measurement and analysis difficult. Thus we treat piecemeal swallowing as a special case and will discuss it later.

**Figure 11:**
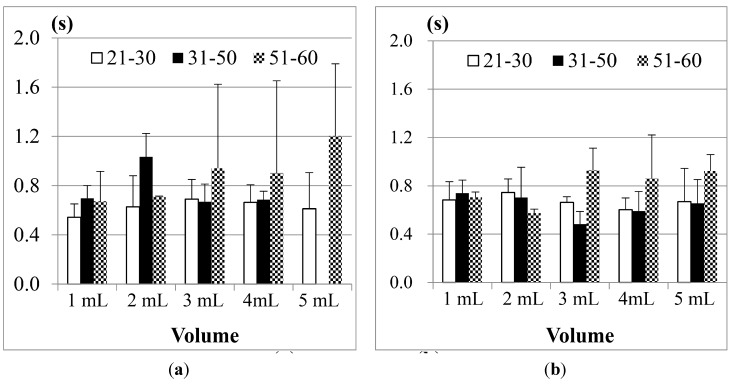
Comparison of the average duration of SAD in different age groups for: (**a**) male; and (**b**) female participants. The X-axis lists the water volumes and the Y-axis shows the mean duration time.

Figure 11 shows the SAD results, calculated from the nasal airflow, where (a) is for male participants; and (b) is for female participants. Here also the 20 mL water results of the male group aged from 31 to 50 years old are not included due to the piecemeal swallowing mentioned above.

**Figure 12:**
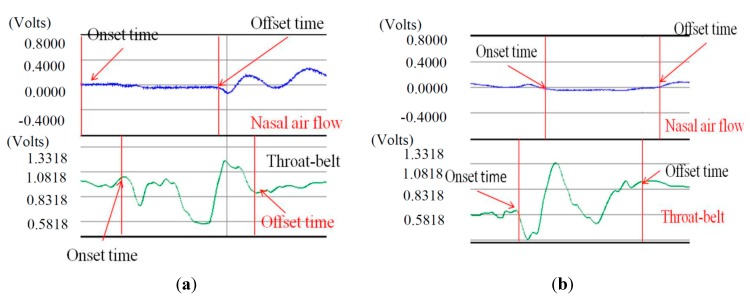
Two cases of SAD: (**a**) the onset time of SAD is earlier than the onset time of FSR; (**b**) the offset time of SAD is later than the time of FSR.

As we can see in Figure 11, the male and the female participants aged from 51 to 60 years old have obviously longer SAD duration in the 5, 10, and 20 mL tests than the other age participants, but for the participants aged from 21 to 30 and from 31 to 50, there are no significant differences for different volumes of water. We know that when the throat pushes the water or bolus into the stomach via the esophagus, the breathing will undergo a brief apnea period. The duration of this period (*i.e.*, SAD in Figure 6) will differ, mostly depending on each one’s swallowing behavior. In our tests, some participants stopped breathing before swallowing for a very short time. Even after swallowing the water, some participants will continue to have this apnea period for a while. Figure 12 illustrates both of these cases if we compare the nasal airflow signals with the FSR signals from our throat belt.

Figure 12a is the case of which the participant is a 21 year-old male in the 5 mL test. The nasal air flow signals show that the onset time of SAD (*i.e.*, apnea period) is earlier than the onset time of FSR (*i.e.*, the time of the thyroid cartilage starts to move). Different from Figure 12a, Figure 12b is another case of which the subject is a 40 year-old male in the 10 mL test. In this case, the offset time of SAD happens later than the time the thyroid cartilage stops the motions. This is why in in Figure 11a there are some cases where the standard deviations are particularly large. In our testing, we find that the swallowing function cannot be distinguished clearly only by sEMG or only by SAD. Thus in the next section, we will add the FSR throat belt to the testing for swallowing function evaluation.

#### 4.1.2. The Results of Jitter and TET by the Throat Belt

Recall that the duration of Jitter and TET (in Figure 6) can be used to measure the motions of the thyroid cartilage. According to the research of Wang and Chuang *et al.* [21,23], the duration of Jitter and TET will be affected by the bolus volume. Here we use the FSR throat belt to measure Jitter and TET, and compare the effects of the bolus volumes on different genders and age groups.

(1) Comparison between men and women

**Figure 13:**
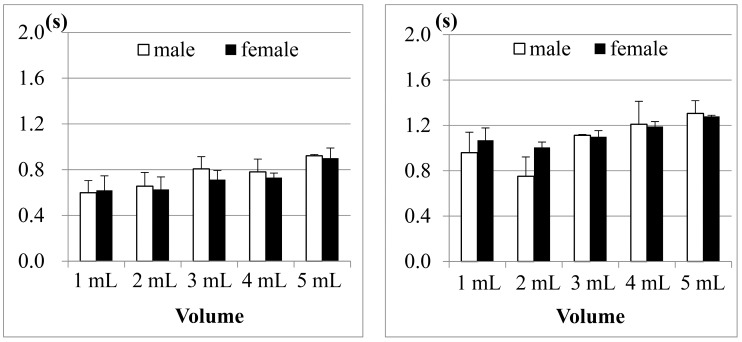
Average duration of (**a**) Jitter and (**b**) TET for the age group 21–30 years old.

Figure 13a shows the results of Jitter, and Figure 13b shows the results of TET for the participants aged from 21 to 30 years old in tests with different water volumes. The results show that both male and female participants have longer Jitter and TET durations as the volumes of water increase, except for the case of 1 mL of water. The reason is the same as what we mentioned in Figure 10.

The results in Figure 13 also show that both the Jitter and TET of young male participants (aged from 21 to 30), on average, are slightly longer than those of the female participants (aged from 21 to 30) when the volume of water is larger than 5 mL, but for the volumes smaller than 5 mL, the male participants seem to have shorter Jitter and TET. We use pairwise *T*-test to evaluate if the male and female participants in this age group have obvious differences (*i.e.*, research hypothesis). Table 2 shows the statistical analysis of average Jitter and average TET between the male and female groups aged from 21 to 30 years old for different water volumes. The research hypothesis will be accepted if (*T*-value > *F*-value) or (*T*-value < −*F*-value) at the α = 0.05 level. From Table 2 we can find that in this age group of the participants, the average Jitter or TET between different genders do not have statistically significant differences.

**Table 2:** Statistical analysis of average Jitter and average TET between the male and female groups aged from 21 to 30 years old for different water volumes.

(α= 0.05)		1 mL		3 mL		5 mL		10 mL		20 mL	
		Jitter	TET	Jitter	TET	Jitter	TET	Jitter	TET	Jitter	TET
Male’s mean		0.599	0.959	0.656	0.751	0.807	1.112	0.781	1.210	0.921	1.305
Female’s mean		0.620	1.068	0.627	1.006	0.713	1.100	0.731	1.190	0.900	1.280
95% CI *	Upper	0.876	1.285	0.740	1.365	0.804	1.162	0.816	1.332	0.989	1.368
	Lower	0.573	1.017	0.593	0.913	0.678	1.046	0.695	1.076	0.861	1.205
Degree of freedom (*df)*		13	13	16	16	12	12	9	9	7	7
*F*-value		2.160	2.160	2.120	2.120	2.179	2.179	2.262	2.262	2.365	2.365
*T*-value		−0.609	−1.790	1.156	−1.257	1.380	0.199	1.053	0.117	0.221	0.079
*p-*value		0.552	0.097	0.265	0.226	0.193	0.846	0.319	0.909	0.831	0.939
Cohen’s d (Effect size)		−0.321	−0.943	0.545	−0.577	0.770	0.111	0.638	0.071	0.148	0.053

* CI = Confidence Interval of this age group.

**Figure 14:**
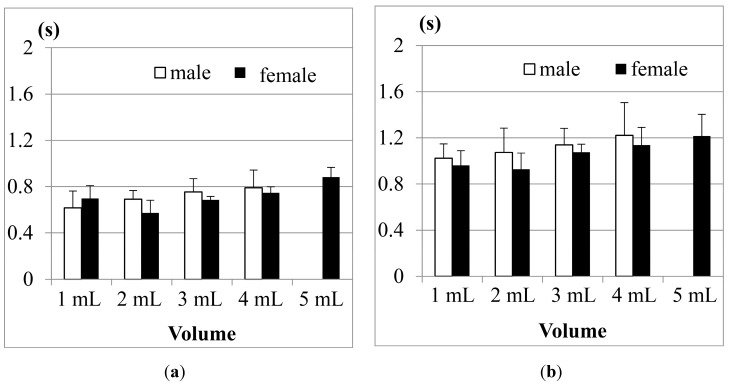
Average duration of (**a**) Jitter and (**b**) TET for the age group 31–50 years old.

Figure 14 further shows the results of average Jitter and average TET for the 31–50 years old age group. We can find that Jitter and TET become longer as the volume of water increases. Moreover, when we increase the volume of water to 20 mL, the male participants display piecemeal swallowing situations. In this case, they need to swallow the 20 mL water in many times, therefore their Jitter and TET cannot be measured.

For this age group (31–50), we also find that the male participants in most measurements need longer Jitter and TET durations to finish the swallowing than the female participants. We did pairwise T-tests to compare the male and female groups. Table 3 shows the statistical analysis of average Jitter and average TET between the male and female groups aged from 31 to 50 years old for different water volumes. We can find that in the 3 mL and 5 mL trials, the average Jitter shows a statistical difference (*i.e.*, *T*-value > *F*-value), while the average TET does not. This result is consistent with the study in [24], where they found that the male participants performed slightly worse than the female participants in this age group.

**Table 3:** Statistical analysis of average Jitter and average TET between the male and female groups aged from 31 to 50 years old for different water volumes.

(α = 0.05)		1 mL		3 mL		5 mL		10 mL		20 mL	
		Jitter	TET	Jitter	TET	Jitter	TET	Jitter	TET	Jitter	TET
Male’s mean		0.616	1.023	0.691	1.073	0.754	1.139	0.789	1.221	N/A	N/A
Female’s mean		0.698	0.962	0.573	0.928	0.686	1.076	0.746	1.137	0.883	1.215
95% CI *	Upper	0.698	1.032	0.679	1.088	0.764	1.201	0.805	1.277	0.914	1.237
	Lower	0.573	0.905	0.550	0.921	0.682	1.054	0.701	1.042	0.852	1.074
Degree of freedom (*df*)		18	18	14	14	19	19	17	17	19	19
*F*-value		2.101	2.101	2.145	2.145	2.093	2.093	2.110	2.110	2.093	2.093
*T*-value **		−1.763	0.947	**5.147**	1.481	**2.424**	1.800	0.533	0.304	N/A	N/A
*p-*value		0.095	0.356	**0.001**	0.156	**0.025**	0.088	0.601	0.765	N/A	N/A
Cohen’s d (Effect size)		−0.788	0.424	2.658	0.662	1.069	0.794	0.245	0.139	N/A	N/A

* CI = Confidence Interval; ** The bold number means that *T*-value > *F*-value.

**Figure 15:**
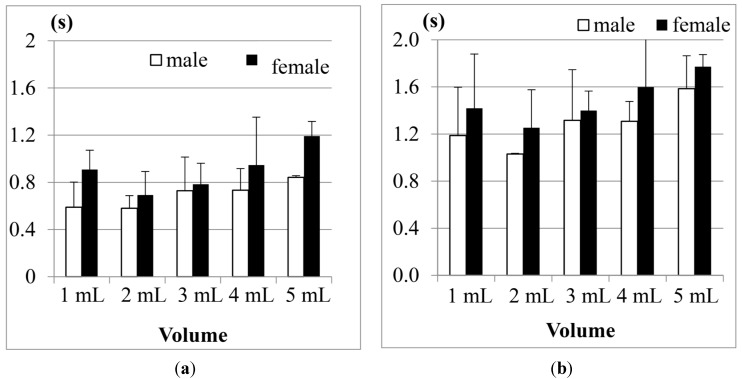
Average duration of (**a**) Jitter and (**b**) TET for the age group 51–60 years old.

Finally, the results of the 51–60 year old age group are shown in Figure 15. Compared with the previous two age groups, the participants aged from 51 to 60 obviously needed even longer durationd of Jitter and TET to finish swallowing in all measurements. Particularly, in this age group, we note that the female participants on average need longer Jitter and TET durationd than the male participants, which is quite different from the results in the two previous age groups.

If we apply a pairwise T-test to compare the male and female participants in this age group (51–60), we find that the durations of Jitter in the 1, 10 and 20 mL measurements show more significant differences (*i.e.*, *T*-value < −*F*-value) than the durations of TET (see Table 4). From Figure 13, Figure 14 and Figure 15, our testing suggests that the measurement of Jitter can have more distinctive sensitivity than the measurement of TET.

**Table 4:** Statistical analysis of average Jitter and average TET between the male and female groups aged from 51 to 60 years old for different water volumes

		1 mL		3 mL		5 mL		10 mL		20 mL	
		Jitter	TET	Jitter	TET	Jitter	TET	Jitter	TET	Jitter	TET
Male’s mean		0.589	1.187	0.580	1.029	0.728	1.316	0.733	1.307	0.842	1.584
Female’s mean		0.908	1.418	0.693	1.253	0.784	1.399	0.946	1.598	1.192	1.771
95% CI *	Upper	0.904	1.524	0.732	1.302	0.917	1.579	1.096	1.754	1.234	1.852
	Lower	0.594	1.081	0.538	1.001	0.603	1.135	0.660	1.278	0.917	1.566
Degree of freedom (*df*)		10	10	9	9	7	7	7	7	7	7
*F*-value		2.228	2.228	2.262	2.262	2.365	2.365	2.365	2.365	2.365	2.365
*T*-value **		**−3.004**	−1.216	−1.615	−1.889	-0.645	−0.592	**−2.725**	**−2.746**	**−4.099**	**−5.651**
*p-value*		**0.013**	0.252	0.141	0.091	0.539	0.572	**0.029**	**0.028**	**0.004**	**0.001**
Cohen’s d (Effect size)		−1.734	−0.702	−0.978	−1.144	−0.456	−0.419	−1.828	−1.942	−2.898	−3.996

* CI = Confidence Interval; ** The bold number means that *T*-value < −*F*-value.

(2) Effects of the Age

The authors in the study [18,19] mentioned that age will have more impact on the swallowing ability; when the age increases, the duration of Jitter and TET will become longer. According to this point, we rearrange the data and compare the swallowing ability among different age groups.

**Figure 16:**
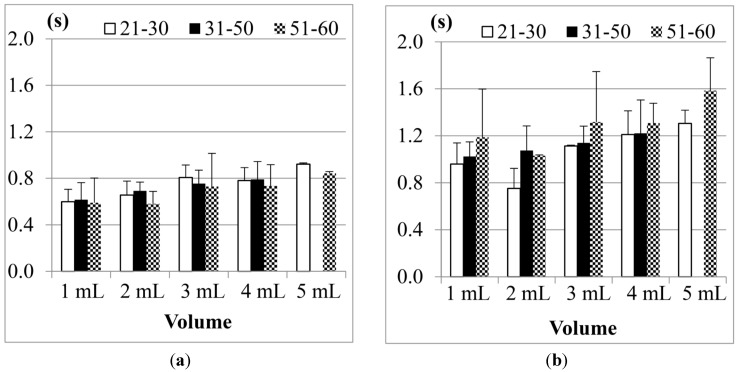
Average results of the male participants in different age groups for (**a**) Jitter and (**b**) TET.

Figure 16 shows the results of Jitter and TET for the male participants in three age groups (the data of the participants aged from 31 to 50 years old in the 20 mL measurement was not included due to the piecemeal swallowing).

We find that the participants aged from 51 to 60 years old, on average, need the longest TET (total swallowing time) but the shortest Jitter (the duration of the thyroid cartilage pushing the bolus into the esophagus) to finish the swallowing of water, compared with the other two age groups. This shows that the overall swallowing ability of the elder participants is not as good as the younger participants such that their swallowing needs longer time in the pharyngeal stage. On the other hand, the strength of the thyroid cartilage for the elder participants may be not so good, too. That is why the water bolus will fall into the esophagus in a shorter period, which could potentially bring higher risks of choking injuries.

**Figure 17:**
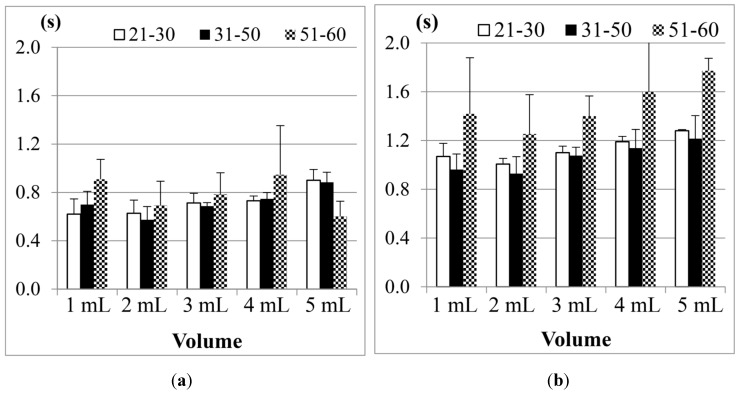
Average results of the female participants in different age groups for (**a**) Jitter and (**b**) TET.

Figure 17 showed the results of the female participants. As we can see, the female participants aged from 51 to 60 years old also have the longest TET but shorter Jitter, even worse than the male participants. It shows that both of their swallowing function of the thyroid cartilage could deteriorate at a certain rate at this age, but the female participants in this age group may have a higher risk of choking injuries than the male group in their daily eating.

We would like to know if the results shown in Figure 16 and Figure 17 have statistical meaning. Therefore we use Analysis of Variance (ANOVA) testing to compare the three different age groups (*i.e.*, 21–30, 31–50, and 51–60). Table 5 shows the testing results for Jitter, and Table 6 for TET. In both tables we list the degree of freedom and the sum of squares for each trial. Also, *F*-values are calculated and compared with *F*-boundaries at the α = 0.05 level. If (*F*-value > *F*-boundary) or (*F*-value < −*F*-boundary), the null hypothesis (*i.e.*, those three age groups did not have obvious difference) can be rejected. Finally the values of “eta squared” are listed in the last row for reference.

From Table 5, we find that the parameter “Jitter” does not show significant differences between the different age groups in most water volumes except 20 mL because their *F*-values were not large enough. From Table 6, however, the “TET” parameter shows totally different results. In those five different water volumes, we have up to four *F*-values (*i.e.*, 1, 5, 10, and 20 mL) larger than *F*-boundaries. It shows that these three age groups really have a statistical difference in the total swallowing time (TET).

**Table 5:** ANOVA testing of “Jitter” for three different age groups in different water volumes.

ANOVA of Jitter	1 mL	3 mL	5 mL	10 mL	20 mL
Between groups *df* *	2	2	2	2	2
Within groups *df* *	44	42	41	36	25
*F*-boundary (α = 0.05)	3.20	3.22	3.22	3.26	3.39
*F*-value **	1.192	0.754	0.255	1.841	**15.818**
*p*-value	0.313	0.477	0.776	0.173	**0.001**
eta squared η^2^	0.051	0.035	0.012	0.093	0.559

* Degree of freedom; ** The bold number means that (*F*-value > *F*-boundary).

**Table 6:** ANOVA testing of “TET” for three different age groups in different water volumes.

ANOVA of TET	1 mL	3 mL	5 mL	10 mL	20 mL
Between groups *df* *	2	2	2	2	2
Within groups *df* *	44	42	41	36	25
*F*-boundary (α = 0.05)	3.20	3.22	3.22	3.26	3.39
*F*-value **	**7.086**	1.073	**5.864**	**6.166**	**7.854**
*p*-value	**0.002**	0.350	**0.005**	**0.004**	**0.002**
eta squared η^2^	0.243	0.044	0.222	0.255	0.428

* Degree of freedom; ** The bold number means that (*F*-value > *F*-boundary).

Next, we are interested about which age group has an obvious mean difference of TET from the other two age groups. Thus we apply the Scheffé *post hoc* test to Table 6 for comparing any two age groups. Table 7, Table 8, Table 9 and Table 10 show the *post hoc* test results of TET for the water volumes of 1, 5, 10 and 20 mL, respectively. In each table, if the 95% confidence interval includes 0, the mean difference (μ*_i_* − μ*_j_*) cannot be considered a significant result. As we can see, the 51–60 year-old age group has obvious mean differences (*i.e.*, the bold numbers) from the other two age groups.

**Table 7:** *Post hoc* test of TET for 1 mL water volume.

Post Hoc Test (1 mL)	Age group (*i*)	Age Group (*j*)	Mean Difference (μ*_i_* − μ*_j_*)	95% Confidence Interval	
				Lower	Upper
Scheffé’s *	51–60	21–30	0.152	−0.077	0.381
	51–60	31–50	**0.334**	0.118	0.551
	21–30	31–50	0.183	−0.020	0.385

* The bold number means that the 95% confidence interval did not include 0.

**Table 8:** *Post hoc* test of TET for 5 mL water volume.

Post Hoc Test (5 mL)	Age Group (*i*)	Age Group (*j*)	Mean Difference (μ*_i_* − μ*_j_*)	95% Confidence Interval	
				Lower	Upper
Scheffé’s *	51–60	21–30	**0.253**	0.061	0.445
	51–60	31–50	**0.229**	0.051	0.408
	21–30	31–50	−0.023	−0.178	0.131

* The bold number means that the 95% confidence interval did not include 0.

**Table 9:** *Post hoc* test of TET for 10 mL water volume.

Post Hoc Test (10 mL)	Age Group (*i*)	Age Group (*j*)	Mean difference (μ*_i_* − μ*_j_*)	95% Confidence Interval	
				Lower	Upper
Scheffé’s *	51–60	21–30	**0.312**	0.035	0.589
	51–60	31–50	**0.357**	0.108	0.607
	21–30	31–50	0.045	−0.189	0.278

* The bold number means that the 95% confidence interval did not include 0.

**Table 10:** *Post hoc* test of TET for 20 mL water volume.

Post Hoc Test (20 mL)	Age Group (*i*)	Age Group (*j*)	Mean Difference (μ*_i_* − μ*_j_*)	95% Confidence Interval	
				Lower	Upper
Scheffé’s *	51–60	21–30	**0.423**	0.051	0.795
	51–60	31–50	**0.554**	0.182	0.926
	21–30	31–50	0.131	−0.241	0.503

* The bold number means that the 95% confidence interval did not include 0.

Therefore we can conclude that the factor of age actually has more significant impact on the swallowing ability than the gender factor. The results also suggest that after 50 years of age, no matter whether men or women are considered, the swallowing ability could deteriorate very obviously.

#### 4.1.3. Piecemeal Swallowing

Some researchers have pointed that a 20 mL bolus volume would be the limit for human swallowing [13,25]. In our measurements, some participants also displayed instances of piecemeal swallowing. Table 11 shows the frequency of piecemeal swallowing in the different age groups in our measurements. We can find that in total 57 measurements, or about 42.1%, are cases of piecemeal swallowing, especially for the male participants after 31 years of age. If this is the case, the 20 mL water bolus could be used as an indicator to detect the deterioration of the swallowing in aging. This is still an open problem. More evidence and experiments are required to verify this.

**Table 11:** Frequency of the Piecemeal Swallowing.

Age	Gender	Number of the Participants	Total Trials	Piecemeal Swallowing of 20 mL	The Frequency of Piecemeal Swallowing
21–30	Male	4	12	0	0%
21–30	Female	3	9	3	33%
31–50	Male	4	12	12	100%
31–50	Female	4	12	6	50%
51–60	Male	2	6	3	50%
51–60	Female	2	6	0	0%
Total		19	57	24	42.1%

#### 4.1.4. Discussion

From the results described above, we conclude that the FSR throat belt can effectively measure and differentiate the swallowing parameters, Jitter and TET, between different age groups for different volumes of water. Compared with other two parameters sEMG and SAD (nasal air flow), we show that the parameters Jitter and TET have better sensitivity for evaluating the swallowing status. By our testing, we also show that a 20 mL volume would be a swallowing limitation. This fact is very important and useful in developing related healthcare applications [26,27]. Also, we show that differences indeed exist between male and female participants, and a deterioration of the swallowing ability will occur with increasing age.

Currently instrument-based approaches (e.g., VFSS) have advantages and limitations. Umay *et al.* recommended that bedside tests should be used mainly as initial screening tests [26]. Therefore the instrument-based approaches should be performed in patients who are at risk for swallowing disorders [26,27]. They are advantageous because they can detect aspiration [28,29], which aids in the clinical management planning of tube or oral feeding. Nonetheless, the disadvantages of radiation exposure and non-portability make it difficult to apply them in cases of acute stage disease, sickness, in large studies, and for repeated measurements within a short period in follow-up studies. In this paper we use a non-invasive assessment method that does not cause any stress to patients or pose a radiation risk. Furthermore, it should be easily carried or worn, and portable. Equally important, this non-invasive swallowing study tool should be capable of being combined with respiration monitoring for swallowing and respiration coordination studies.

**Figure 18:**
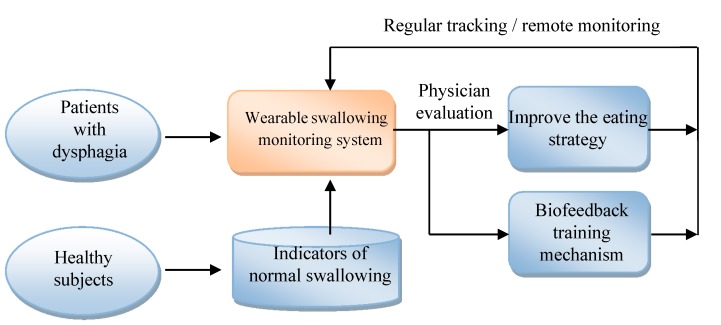
The applications of the wearable dysphagia monitoring system.

Figure 18 shows the applications that can be developed by the system proposed in this paper. At first, this system can be applied to healthy subjects for building the indicators of normal swallowing patterns. Then we can use this monitoring system to measure the swallowing motions of dysphagia patients. The results can be evaluated by the physicians for eating strategy adjustment or for biofeedback training. All above steps can be repeated for regular tracking and monitoring.

**Figure 19:**
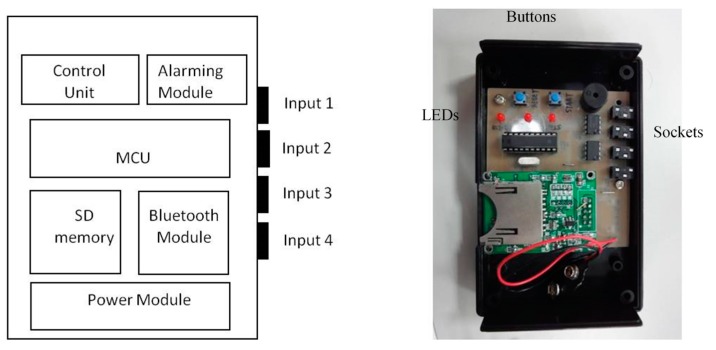
Design of the holter.

We can also connect the FSR throat belt with a holter to develop portable applications. The holter we implemented is shown in Figure 19. It includes four input signal sockets, two control buttons, three output LEDs and one signal processing circuit. To unify the connection between the holter and different kinds of sensors (*i.e.*, FSR, sEMG, nasal airflow *etc.*), we use 3.5 mm stereo connectors as the input port and refit the sensor output pins with those connectors. In our holter design, we reserved four input ports for future possible connections to other sensors for multivariate data measurements. The signal processing circuit is composed of a microcontroller (MCU), a Bluetooth module, a SD memory module and a power-supply module. The MCU we used is an AT89C2051 [30], which is a low-power but high performance chip. We use it to do the digital signal preprocessing operations. The data measured by the sensors can be collected on the SD memory card, or be transferred to a mobile phone via the Bluetooth module.

## 5. Conclusions

In our study, we use the FSR sensor to develop a throat belt to measure swallowing ability. We use different water bolus volumes to observe the swallowing ability differences between male and female participants in different age groups. The experiment results show that the FSR sensor can effectively measure the motions of the thyroid cartilage during swallowing.

We also use this belt to develop a portable holter and an automatic data analysis program. This program can analyze the onset time and the offset time from sEMG, SAD (nasal air flow), and throat belt signals in a synchronization presentation. The functions and the precision of the throat-belt are proven by real measurements. We show that this throat belt design would be an easy-to-use, objective, and non-invasive measurement or assessment tool for developing dysphagia-related services.

While this work focuses on measurements on the healthy participants, in the future, we plan to apply the proposed system to measure other important swallowing indicators for particular diseases. Take the work in [31] for example. They used the acoustic monitoring to measure the frequency of spontaneous swallowing for Parkinson’s disease. The limitation of this approach, however, was the need for a quiet environment to record the sounds of swallowing. Using our FSR-based approach, on the other hand, one could perform such monitoring for is under investigation.
